# Optimizing odor identification testing as quick and accurate diagnostic tool for Parkinson's disease

**DOI:** 10.1002/mds.26637

**Published:** 2016-05-09

**Authors:** Philipp Mahlknecht, Raimund Pechlaner, Sanne Boesveldt, Dieter Volc, Bernardette Pinter, Eva Reiter, Christoph Müller, Florian Krismer, Henk W. Berendse, Jacobus J. van Hilten, Albert Wuschitz, Wolfgang Schimetta, Birgit Högl, Atbin Djamshidian, Michael Nocker, Georg Göbel, Arno Gasperi, Stefan Kiechl, Johann Willeit, Werner Poewe, Klaus Seppi

**Affiliations:** ^1^ Department of NeurologyMedical University InnsbruckInnsbruckAustria; ^2^ Sobell Department of Motor Neuroscience and Movement DisordersUCL Institute of NeurologyLondonUnited Kingdom; ^3^ Department of Neurology, Neuroscience Campus AmsterdamVU University Medical CentreAmsterdamThe Netherlands; ^4^ Divisions of Human NutritionWageningen UniversityWageningenThe Netherlands; ^5^ Study Center Confraternitaet‐PKJ ViennaViennaAustria; ^6^ Department of NeurologyLeiden University Medical CentreLeidenThe Netherlands; ^7^ Department of Applied Systems Research and StatisticsJohannes Kepler University LinzLinzAustria; ^8^ Department of Medical Statistics, Informatics and Health EconomicsMedical University InnsbruckInnsbruckAustria; ^9^ Department of NeurologyHospital of BruneckBruneckItaly

**Keywords:** Parkinson's disease, parkinsonism, tremor, diagnosis, olfactory dysfunction

## Abstract

**Introduction:**

The aim of this study was to evaluate odor identification testing as a quick, cheap, and reliable tool to identify PD.

**Methods:**

Odor identification with the 16‐item Sniffin' Sticks test (SS‐16) was assessed in a total of 646 PD patients and 606 controls from three European centers (A, B, and C), as well as 75 patients with atypical parkinsonism or essential tremor and in a prospective cohort of 24 patients with idiopathic rapid eye movement sleep behavior disorder (center A). Reduced odor sets most discriminative for PD were determined in a discovery cohort derived from a random split of PD patients and controls from center A using L1‐regularized logistic regression. Diagnostic accuracy was assessed in the rest of the patients/controls as validation cohorts.

**Results:**

Olfactory performance was lower in PD patients compared with controls and non‐PD patients in all cohorts (each *P* < 0.001). Both the full SS‐16 and a subscore of the top eight discriminating odors (SS‐8) were associated with an excellent discrimination of PD from controls (areas under the curve ≥0.90; sensitivities ≥83.3%; specificities ≥82.0%) and from non‐PD patients (areas under the curve ≥0.91; sensitivities ≥84.1%; specificities ≥84.0%) in all cohorts. This remained unchanged when patients with >3 years of disease duration were excluded from analysis. All 8 incident PD cases among patients with idiopathic rapid eye movement sleep behavior disorder were predicted with the SS‐16 and the SS‐8 (sensitivity, 100%; positive predictive value, 61.5%).

**Conclusions:**

Odor identification testing provides excellent diagnostic accuracy in the distinction of PD patients from controls and diagnostic mimics. A reduced set of eight odors could be used as a quick tool in the workup of patients presenting with parkinsonism and for PD risk indication. © 2016 The Authors. Movement Disorders published by Wiley Periodicals, Inc. on behalf of International Parkinson and Movement Disorder Society

Olfactory deficits affect 75% to 90% of patients with Parkinson's disease (PD), and olfactory testing may also represent a sensitive screening test for individuals at risk of developing PD,^1^, ^2^, ^3^, ^4^ whereas olfactory function is normal or only mildly impaired in other forms of degenerative parkinsonism or essential tremor (ET).^2^, ^5^ Olfactory testing has recently been incorporated in the newly established International Parkinson and Movement Disorder Society criteria for PD^6^ and prodromal PD.^7^

To test for olfactory performance in PD, most studies have focused on odor identification using the disposable University of Pennsylvania Smell Identification Test (UPSIT) or the reusable Sniffin' Sticks test battery assessing olfactory threshold and odor discrimination in addition to odor identification.^2^, ^8^ Both tests are time‐consuming, and olfactory testing is rarely performed in clinical routine. Most of existing shortened versions of odor identification tests were not specifically developed for PD patients, nor were any of these tests properly validated.^9^, ^10^, ^11^, ^12^, ^13^

Hence, we sought to assess the diagnostic value of the 16‐item Sniffin' Sticks identification subtest (SS‐16) as an easy‐to‐use, inexpensive tool. We also aimed to shorten and optimize it to identify both established and early/prodromal PD using a discovery cohort and different validation cohorts.

## Patients and Methods

For the present study, data from a total of 134 PD patients and 46 patients with atypical parkinsonism (23 multiple system atrophy [MSA], 23 progressive supranuclear palsy [PSP]), who participated in three independent prospective, cross‐sectional clinical studies at the Department of Neurology, Innsbruck Medical University (Innsbruck, Austria)^14^, ^15^ and from 336 age‐matched healthy controls (HCs) and 29 subjects with ET from the prospective population‐based Bruneck Study^16^, ^17^ were analyzed (center A). Patients were regularly followed over at least 24 months to reassess their clinical diagnosis, and 4 cases were reclassified as MSA (n = 1) or PSP (n = 3) during clinical follow‐up. PD patients and HCs from center A were randomly split into approximately equal parts. Patients with MSA, PSP, and ET were subsumed as differential diagnoses (DDs) in the validation cohort only (Supporting Fig. 1). Two independent sets of PD patients and HCs were used as additional validation cohorts; 400 PD patients and 150 HCs from the Departments of Neurology of the VU University Medical Centre (Amsterdam, The Netherlands) and the Leiden University Medical Centre (Leiden, The Netherlands) (center B);^18^ and 112 consecutive PD patients and 120 controls recruited by general neurologists in Vienna, Austria (center C). Last, we used a previously described prospective cohort of 24 patients with polysomnography‐confirmed idiopathic rapid eye movement sleep behavior disorder (iRBD),^19^ consecutively recruited at center A. iRBD patients were tested for olfactory function at baseline and followed up for a mean of 6 years in order to detect incident neurodegenerative diseases, in particular, PD. Studies were approved by the local ethics committees. All participants gave written informed consent according to the Declaration of Helsinki.

Olfactory testing was performed with the SS‐16 (Burghart Medizintechnik, Germany) as described elsewhere.^20^ In center C, the Sniffn' Sticks 12‐item odor identification test (SS‐12),^21^ a commercially available, shorter version of the SS‐16 test, was used. Subscores of reduced sets of odors were derived for the present analyses.

Group comparisons between PD patients and controls or DDs were performed with appropriate tests (see table legends). Odor sets predictive of PD were determined in the discovery cohort by L1‐regularized logistic regression implementing the least absolute shrinkage and selection operator (the LASSO)^22^ using the glmnet R package. The performance of full and reduced odor sets in discriminating PD from controls or DDs was gauged using area under the receiver operating characteristic curve (AUC) with respective 95% confidence intervals (95% CI). Performance of full and reduced odor sets is given by conventional measures of diagnostic accuracy. To adjust for the bias in prevalence of PD versus DDs in our pooled cohort from center A, positive predictive values (PPVs) and negative predictive values (NPVs) were modeled for two additional scenarios using published data on the relative prevalence of PD versus DDs (1) as reported in general neurological services and (2) as assumed in specialized movement disorder services.^23^ Furthermore, we evaluated the accuracy of the SS‐16 and its subscores in (1) identifying PD in cohort A after excluding patients with >3 years of disease duration and (2) predicting incident PD among the 24 idiopathic RBD patients. SPSS (version 22.0; IBM Corp., Armonk, NY) and R software (version 3.2.2; R Foundation for Statistical Computing, Vienna, Austria) were used for statistical analyses. The local significance level was set at *P* < 0.05. Full methods can be found in the Supporting Appendix.

## Results

**Figure 1:**
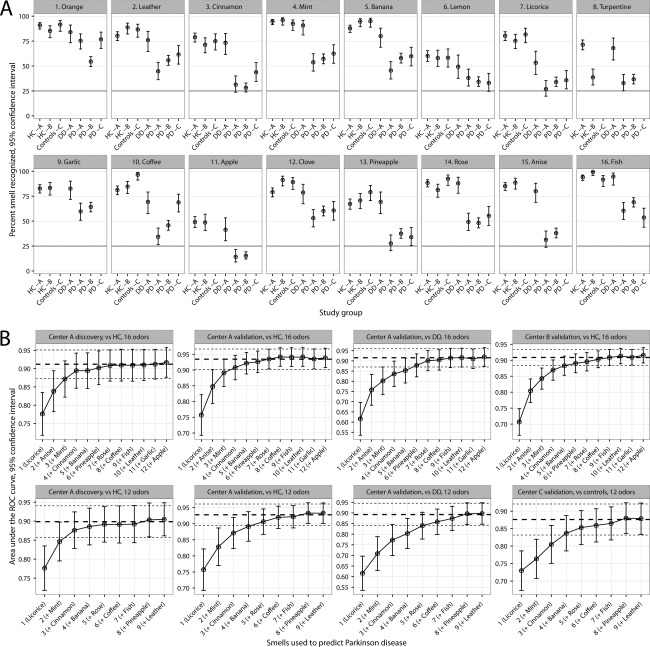
Identification of individual odors in the study groups in the three different centers (A). Gray horizontal line indicates the probability of correctly guessing an odor in the employed forced choice test. AUCs with 95% CIs as a function of combination of odors best predicting PD according to the LASSO analysis in the various cohorts (B). The three horizontal lines in each graph represent the AUCs with 95% CIs of the full test used (SS‐16 for the upper row and SS‐12 for the lower row). The best eight discriminating odors derived from the full SS‐16 were used for the SS‐8 subscore (licorice, anise, mint, cinnamon, banana, pineapple, rose, and coffee).

Characteristics of the patients and controls in the different cohorts are shown in Table 1A and in the Supporting Information. Figure 1A and Supporting Table 1 depict differences in identifying individual odors in the study groups.

**Table 1:** • • •

A: Characteristics of the groups											
	Center A (Innsbruck and Bruneck)							Centre B (Leiden)		Centre C (Vienna)	
	HCs = 336	PD = 134	DDs = 75	MSA = 23	PSP = 23	ET = 29	iRBD = 24	HCs = 150	PD = 400	Controls = 120	PD = 112
Age^a^	68.8 ± 8.3 *P* = 0.99	68.0 ± 8.8	68.8 ± 9.7 *P* = 0.99	63.3 ± 8.9 *P* = 0.090	67.2 ± 6.2 *P* = 0.99	74.5 ± 9.8 *P* = 0.012	66.0 ± 5.0	59.2 ± 7.4 *P* = 0.022	61.4 ± 9.9	67.4 ± 10.4 *P* = 0.54	69.2 ± 8.6
Female (%)^b^	53.6 *P* = 0.003	37.3	48.0 *P* = 0.35	52.2 *P* = 0.75	30.4 *P* = 0.99	58.6 *P* = 0.17	12.5	42.0 *P* = 0.38	37.5	58.3 *P* = 0.24	50.0
Disease duration (yr)^a^	NA	6.2 ± 4.8	8.0 ± 14.1 *P* = 0.99	4.2 ± 3.2 *P* = 0.081	3.1 ± 2.0 *P* = 0.006	18.7 ± 18.0 *P* < 0.001	0.8 ± 1.3	NA	11.4 ± 6.3	NA	6.8 ± 5.4
H & Y^a^	NA	2.4 ± 0.9	3.2 ± 0.7 *P* < 0.001	3.3 ± 0.9 *P* < 0.001	3.0 ± 0.7 *P* = 0.002	NA	NA	NA	2.6 ± 0.8	NA	2.0 ± 0.7
UPDRS‐III^a^	NA	31.3 ± 15.1	36.9 ± 11.5 *P* = 0.002	43.0 ± 9.2 *P* < 0.001	30.9 ± 10.4 *P* = 0.99	NA	2.7 ± 2.9				
MMSE^a^	28.5 ± 1.5 *P* = 0.99	28.8 ± 1.3	27.7 ± 2.2 *P* = 0.018	27.2 ± 2.4 *P* = 0.60	27.0 ± 2.1 *P* = 0.003	28.6 ± 1.7 *P* = 0.99			27.4 ± 2.6		
SS‐12 Sum^a^	9.8 ± 2.1 *P* < 0.001	5.4 ± 2.5	9.1 ± 1.9 *P* < 0.001	9.1 ± 1.6 *P* < 0.001	8.4 ± 2.1 *P* < 0.001	9.5 ± 1.9 *P* < 0.001				10.3 ± 1.9 p<0.001	6.5 ± 2.7
SS‐16 Sum^a^	12.7 ± 2.7 *P* < 0.001	6.8 ± 3.1	11.8 ± 2.4 *P* < 0.001	11.7 ± 2.1 *P* < 0.001	10.9 ± 2.6 *P* < 0.001	12.6 ± 3.7 *P* < 0.001	9.9 ± 4.4	12.6 ± 2.3 *P* < 0.001	7.4 ± 3.0		

B: Diagnostic accuracy of the SS‐16 and the SS‐8 in the identification of PD in the various cohorts									
	Set of Odors	AUC (95% CI)	Cutoff	Sensitivity (95% CI)	Specificity (95% CI)	Specificity vs. MSA	Specificity vs. PSP	Specificity vs.ET	Accuracy (95% CI)
Discovery: PD vs. HC (center A)	SS‐16	0.91 (0.87–0.95)	≤10	85.9% (75.8–92.4)	86.1% (80.1–90.6)				86.1% (81.1–89.9)
	SS‐8	0.91 (0.87–0.95)	≤5	88.7% (79.1–94.4)	84.4% (78.2–89.1)				85.7% (80.7–89.5)
Validation: PD vs. HC (center A)	SS‐16	0.93 (0.90–0.97)	≤10	92.1% (82.3–97.0)	86.5% (80.3–91.0)				88.5% (83.1–91.7)
	SS‐8	0.94 (0.91–0.97)	≤5	93.7% (84.3–98.0)	84.0% (77.6–88.9)				86.7% (81.6–90.6)
Validation: PD vs. DD (center A)	SS‐16	0.92 (0.87–0.96)	≤10	92.1% (82.3–97.0)	76.0% (65.1–84.3)	78.3% (57.7–90.8)	65.2% (44.8–81.3)	82.8% (65.0–92.9)	83.3% (76.2–88.7)
			≤9^c^	84.1% (73.0–91.3)	84.0% (73.9–90.8)	87.0% (67.0–96.3)	73.9% (53.2–87.7)	89.7% (72.8–97.2)	84.1% (77.0–89.3)
	SS‐8	0.91 (0.85–0.96)	≤5	93.7% (84.3–98.0)	72.0% (60.9–81.0)	69.6% (48.9–84.6)	69.6% (48.9–84.6)	75.9% (57.6–88.1)	81.9% (74.6–87.5)
			≤4^c^	84.1% (73.0–91.3)	88.0% (78.5–93.8)	91.3% (72.0–98.8)	78.3% (57.7–90.8)	93.1% (77.0–99.2)	86.2% (79.4–91.1)
Validation: PD vs. HC (center B)	SS‐16	0.91 (0.88–0.93)	≤10	83.3% (79.3–86.6)	82.0% (75.0–87.4)				82.6% (79.1–85.5)
	SS‐8	0.90 (0.88–0.93	≤5	85.3% (81.4–88.4)	83.3% (76.5–88.5)				84.7% (81.5–87.5)

Part A of the table: *P* values report significances of comparisons of values in respective columns/groups versus PD within centers (A, B, and C) and are post hoc Bonferroni corrected for center A. Part B of the table: Preferred cutoffs of predictive scores were determined by Youden's index in the discovery cohort and, in a subgroup analysis, did not differ between sexes. SS‐8 = subscore of the eight best‐discriminating odors (licorice, anise, mint, cinnamon, banana, pineapple, rose, and coffee).

a Results represent means ± standard deviation; *P* values calculated using Mann‐Whitney's U test.

b *P* value calculated using chi‐square test.

c Additional lower cutoffs were applied in the distinction versus DDs because a mildly decreased sense of smell had been reported in MSA, PSP, and ET patients^2^, ^5^ and our model was established in a comparison of PD patients with HCs (discovery cohort).

MMSE, Mini–Mental State Examination; NA, not applicable.

An increasing discriminatory power in the distinction of PD patient versus HC, as demonstrated in AUCs, was achieved with an increasing number of odor items used in the discovery cohort (Fig. 1B). This could be reproduced in the validation sets, reaching the 95% confidence interval (CI) of AUCs achieved with the entire Sniffin Sticks tests (SS‐16 and SS‐12; upper and lower row in Fig. 1B, respectively) when using only six sticks and the optimum when using eight (SS‐8). We assessed diagnostic accuracy of the SS‐16 and SS‐8 in identifying PD patients (Table 1B). Of note, all 4 patients who were reclassified (MSA, 1 case; PSP, 3 cases) during clinical follow‐up had a normal olfactory function at baseline according to the SS‐16 and SS‐8. In a modeled general neurological service (PD prevalence: 91.8%), both the SS‐16 and the SS‐8 would yield PPVs of >97%. In a specialized outpatient clinic (lower PD prevalence 69.0% because of higher proportion of non‐PD parkinsonism), PPVs of around 90% would be achieved (Supporting Table 2). To test the usefulness of the SS‐16 and the SS‐8 as a screening method for early/prodromal PD, we repeated the diagnostic accuracy analyses after excluding patients with >3 years of disease duration, which did not alter the results (Supporting Table 3). Furthermore, the 8 incident PD cases among iRBD patients were predicted with the SS‐16 and the SS‐8 with the same sensitivity of 100.0% (95% CI: 62.8–100.0), specificity of 68.8% (95% CI: 44.2–86.1), PPV of 61.5% (95% CI: 35.4–82.4), and NPV of 100.0% (95% CI: 70.0–100.0).

## Discussion

We found excellent diagnostic accuracy for the SS‐16 and a shortened test, the SS‐8, in the distinction of PD not only from controls, but also from non‐PD tremor or atypical parkinsonism.

To the best of our knowledge, our study is the largest study of olfactory testing ever performed in patients with PD, related disorders, and controls comprising a total of 1,351 individuals. We employed a sophisticated logistic regression analysis to determine reduced sets of odors along the LASSO regularization path in a discovery cohort. This variable selection algorithm considers the statistical dependencies among odor‐specific olfactory impairments and minimizes redundancy. Whereas the diagnostic performance in identifying PD of the three, four, or five best‐discriminating odors was inferior to the whole SS‐16, the six best discriminating odors achieved accuracy within the 95% CI of the AUCs of the entire set, which was further improved by using a combination of eight odors (but not beyond).

Short tests such as the SS‐8 might be particularly appealing for two purposes: First, in a clinical setting, they might serve as an additional quick (approximately 3 minutes) and handy tool in the workup of patients presenting with parkinsonism where clinicians want to identify true PD cases with a high specificity and predictivity. In our sample, the specificity for PD was high (84% with the SS‐16 and 88% with the SS‐8) combined with a high sensitivity (84%). When modeling prevalences in a general neurological service and a specialized movement disorders outpatient clinic, the PPVs were high at 98% and 94%, respectively. The usefulness of the SS‐16 and SS‐8 for ruling out DDs is further supported by the analysis in parkinsonian patients with less than 3 years of disease duration yielding a similar diagnostic accuracy as in the whole sets. Indeed, all 4 patients in whom an initial diagnosis of PD was later changed to MSA or PSP during follow‐up had a normal olfactory function.

Second, a short olfactory test could be useful as a highly sensitive screening tool in population‐based studies seeking to define cohorts at high risk for PD.^3^, ^24^ Along these lines, we found a high sensitivity of the SS‐16 and SS‐8 in identifying PD versus HCs in the center A (92% and 94%) and center B (83% and 85%) validation cohorts combined with a good specificity of ≥82%. This excellent diagnostic accuracy remained unchanged when only PD patients with less than 3 years of disease duration were included. Furthermore, the SS‐16 and SS‐8 accurately identified 8 incident PD cases from a previously described cohort of 24 iRBD patients clinically followed for 6 years.

Whereas previous studies focused on even shorter sets of three odors in the Sniffin' Sticks or UPSIT,^9^, ^10^, ^11^, ^12^, ^13^ in our analysis six to eight odors emerged as the smallest number with equal performance as the entire set. In line with previous evidence,^25^ this argues against the concept of selective anosmia in PD.^13^ Also, one must take into account that the nature of the Sniffin' Sticks (and the UPSIT) as a forced‐choice test bearing an inherent 25% likelihood of a correct answer, which limits the options of setting cutoffs in reduced odor sets, possibly resulting in unsatisfactory specificity and/or sensitivity. It should be noted that none of the previous studies used independent validation samples, which is a particular strength of our study.

However, there are limitations. Diagnoses of PD and DDs were made according to clinical criteria without pathological confirmation. Therefore, misdiagnosis cannot be ruled out. However, in center A, patients with parkinsonism were followed up for at least 2 years in order to reduce likelihood of misdiagnoses. Furthermore, cultural differences may impact on short olfactory tests to a greater extent compared to longer sets, where a greater variety of odors might balance such effects.^26^ Nevertheless, given the reproducibility shown in the external validation samples, it is likely that diagnostic accuracy in other samples will be similar.

To conclude, our analysis confirms that odor identification testing with the SS‐16 is associated with excellent accuracy in diagnosing PD and shows that it can be shortened considerably without losing diagnostic power. A shortened test of eight odors may be of substantial value in both a clinical setting assisting in the distinction from frequent diagnostic mimics and in a population‐based setting for PD risk evaluation.

## Author Roles

(1) Research Project: A. Conception, B. Organization, C. Execution; (2) Statistical Analysis: A. Design, B. Execution, C. Review and Critique; (3) Manuscript Preparation: A. Writing of the First Draft, B. Review and Critique.

P.M.: 1A, 1B, 1C, 2A, 2B, 2C, 3A, 3B

R.P.: 2A, 2B, 2C, 3B

S.B.: 1B, 1C, 3B

D.V.: 1B, 1C, 3B

B.P.: 1B, 1C, 3B

E.R.: 1B, 1C, 3B

C.M.: 1B, 1C, 3B

F.K.: 1B, 1C, 3B

H.W.B.: 1B, 1C, 3B

J.J.v.H.: 1B, 1C, 3B

A.W.: 1B, 1C, 3B

W.S.: 2A, 2B, 2C, 3B

B.H.: 1B, 1C, 3B

A.D.: 1B, 1C, 3B

M.N.: 1B, 1C, 3B

G.G.: 2A, 2B, 2C, 3B

A.G.: 1B, 1C, 3B

S.K.: 1B, 1C, 3B

J.W.: 1B, 1C, 3B

W.P.: 1B, 1C, 3B

K.S.: 1A, 1B, 1C, 2A, 2B, 2C, 3A, 3B

## Financial Disclosures

Nothing to report.

## Supporting information

Additional Supporting Information may be found in the online version of this article at the publisher's web‐site.

Supplementary Information
