# Supplementary material for: Optimizing odor identification testing as quick and accurate diagnostic tool for Parkinson's disease
Source: Mov Disord. 2016 May 9;31(9):1408–13. doi: 10.1002/mds.26637 (PMC5026160; doi:10.1002/mds.26637)
Supplement: Supplementary file 1 — Supplementary Information [file MDS-31-1408-s001.docx]

**Supplemental Appendix**

**Optimizing odor identification testing as quick and accurate diagnostic tool for Parkinson’s disease**

**METHODS**

**Participants and design of the study**

For the present study data from 134 Parkinson’s disease (PD) patients and 46 patients with atypical parkinsonism (23 multiple system atrophy [MSA], 23 progressive supranuclear palsy [PSP]), who participated in three independent prospective, cross-sectional clinical studies at the Department of Neurology, Innsbruck Medical University, Austria (MFI 6169, ÖNB 14174, and FWF-KLIF KLI82-B00)^1,2^ and from 336 age-matched healthy controls (HC) from the prospective population-based Bruneck Study^3,4^ were analyzed (center A). Clinical diagnoses of MSA, PSP, and PD were made according to the UK-PD-Brain Bank and other current criteria^5–7^ by movement disorder specialists experienced in parkinsonian disorders (K.S. and W.P.). Patients were regularly followed over at least 24 months to re-assess their clinical diagnosis and 4 cases were reclassified as MSA (n=1) or PSP (n=3) during clinical follow-up. Furthermore, 29 subjects with essential tremor (ET) according to the MDS consensus clinical criteria^8^ identified in the Bruneck Study sample were also included.

PD patients and HC from center A were randomly split into approximately equal parts balanced according to diagnosis, age, sex, and total olfactory identification scores, creating a discovery cohort and a validation cohort. These two groups were also not significantly different with regard to other characteristics (disease duration, UPDRS-III scores, H&Y scores, and MMSE scores; all p-values >0.3). Patients with MSA, PSP and ET were subsumed as differential diagnoses (DDs) in the validation cohort only (Supplementary Figure 1).

Two independent sets of PD patients and HC were used as additional validation cohorts: one consisted of 400 PD patients and 150 HC recruited from the outpatient clinics of the departments of Neurology of the VU University Medical Centre and the Leiden University Medical Centre, Netherlands, as previously reported (center B).^9^ The second included 112 consecutive PD patients and 120 controls (patients with neurovascular diseases, n=46; and HC, n=74) recruited in 10 neurological offices by general neurologists with a special interest in movement disorders in Vienna, Austria (center C).

Lastly, in order to perform sensitivity analysis on the predictive value of odor-identification testing for incident PD, we used a previously described prospective cohort of 24 patients with polysomnography-confirmed idiopathic RBD,^10^ consecutively recruited at center A. Idiopathic RBD patients were followed-up for a mean of 6 years in order to detect incident neurodegenerative diseases, in particular PD.

In all cohorts, individuals with potential causes of symptomatic olfactory loss (e.g. head trauma, nasal fracture or surgery, recent or chronic upper airway infection) and individuals with a clinical diagnosis of dementia were not included. The study was approved by the local ethics committees. All participants gave written informed consent according to the Declaration of Helsinki.

**Neurological examination**

All participants underwent a thorough neurological evaluation. In cohort A disease severity was assessed using the motor section of the Unified PD Rating Scale (UPDRS-III) and Hoehn & Yahr (H&Y) scale. Cognitive performance was evaluated by the Mini Mental State Examination (MMSE). In cohorts B and C the H&Y scale was used for scoring disease severity. All PD patients were examined on regular medications.

**Olfactory testing**

The SS-16 consisting of reusable pen-like devices dispensing 16 common odors (Burghart Medizintechnik, Germany) was used to assess olfactory performance in a quiet well-ventilated room. Each odorant was held approximately 2 cm in front of both nostrils for approximately 3 seconds with an interval of 20 to 30 seconds between single odors (the full test takes about 6 to 8 minutes to perform). On a multiple forced-choice task, identification of the odors was performed from a list of 4 descriptors. The total score ranges from 0 to 16 points. In cohort C the Sniffn’ Sticks 12-items odor-identification test (SS-12),^11^ a commercially available, shorter version of the SS-16 test was used. For the present analyses subscores of reduced sets of odors were derived from the SS-16 and the SS-12.

**Statistics**

Group comparisons between PD patients and controls or DDs were performed with appropriate parametric or non-parametric tests and adjustment for age and sex and Bonferroni-correction were applied where appropriate (see table legends).

Odor sets predictive of PD were determined in the discovery cohort by L1-regularized logistic regression implementing the least absolute shrinkage and selection operator (the LASSO)^12^ using the *glmnet* R package.^12^ Sets of increasing size were determined by the sequence in which coefficients became nonzero along the LASSO regularization path. Final number of odors relied on the prior expectation that excellent discrimination could be achieved with approximately half the number of items. Final sets were specified 100% independently of validation data. For each odor-set, predictive scores were calculated as the number of items recognized. The performance of full and reduced odor sets in discriminating PD from controls or DDs was gauged using area under the ROC curve (AUC) with respective 95% confidence intervals (95%CI). Preferred cut-offs of predictive scores were determined by Youden index^13^ and performance of full and reduced odor sets is given by conventional measures of diagnostic accuracy. As a mildly decreased sense of smell has been reported in MSA, PSP, and ET patients^14,15^ and our model was established in a comparison of PD patients with HC, we assumed that lower cut-offs in the distinction of PD versus DDs will be necessary in order to maximize specificity. In addition, to adjust for the prevalence of PD versus DDs in our pooled and therefore artificial cohort A, positive predictive values (PPV) and negative predictive values (NPV) were modeled for two additional scenarios according to published data on the relative prevalence of PD versus DDs 1. as reported in population-based studies and 2. as assumed in specialized movement disorder services.^16^

As a sensitivity analysis, we further tested the usefulness of the SS-16 scores and its subscores as screening method for early/prodromal PD. For this purpose, we evaluated the accuracy in 1. identifying PD in cohort A after excluding patients with >3 years disease duration and 2. in predicting incident PD among the 24 idiopathic RBD patients.

SPSS 22.0 (IBM Corp., Armonk, NY) and R 3.2.2 (R Foundation for Statistical Computing, Vienna, Austria) were used for statistical analyses. The local significance level was set at p<0.05.

**Results**

**Clinical and demographic data**

Characteristics of the patients and controls in the different cohorts are shown in table 1 in the original publication. In cohort A female HC had significantly higher SS-16 scores compared to male HC (13.1 ± 2.6 versus 12.3 ± 2.8; p=0.003), whereas there were no significant sex differences in any of the disease groups. Age was inversely correlated with SS-16 scores in HC (r=-0.36, p<0.001), PSP patients (r=-0.53, p=0.009), and ET patients (r=-0.35, p=0.062), but not in PD or MSA patients. Disease duration was not related to olfactory performance in any of the disease groups. Severity of the disease as measured by the UPDRS-III and the H&Y scale correlated with decreasing SS-16 scores only in the MSA group (r=-0.47, p=0.023; and r=-0.48, p=0.020), but not in PSP or PD patients. In cohort B, SS-16 scores were correlated to age in PD patients (r=-0.36, p<0.001), but not in HC. There were no sex differences in controls. In PD patients, females outperformed males in olfactory scores (8.3±3.2 versus 6.8±2.7; p<0.001), whereas there was no effect of disease duration or disease severity.^9^ In cohort C, SS-12 scores were correlated to age in controls (r=-0.43, p<0.001), but not in PD patients. Among PD patients, females had non-significantly higher SS-12 scores compared to males (7.0±2.8 versus 6.0±2.5; p=0.052), whereas there were no sex differences in controls. While disease duration was not related to SS-12 scores in PD patients, severity of the disease as measured by H&Y scale was associated with decreased olfactory performance (r=-0.23, p=0.015). PD patients had lower SS-16 (and/or SS-12) scores compared to HC in all cohorts, and compared to any other disease group in cohort A (all p-values<0.001).

**
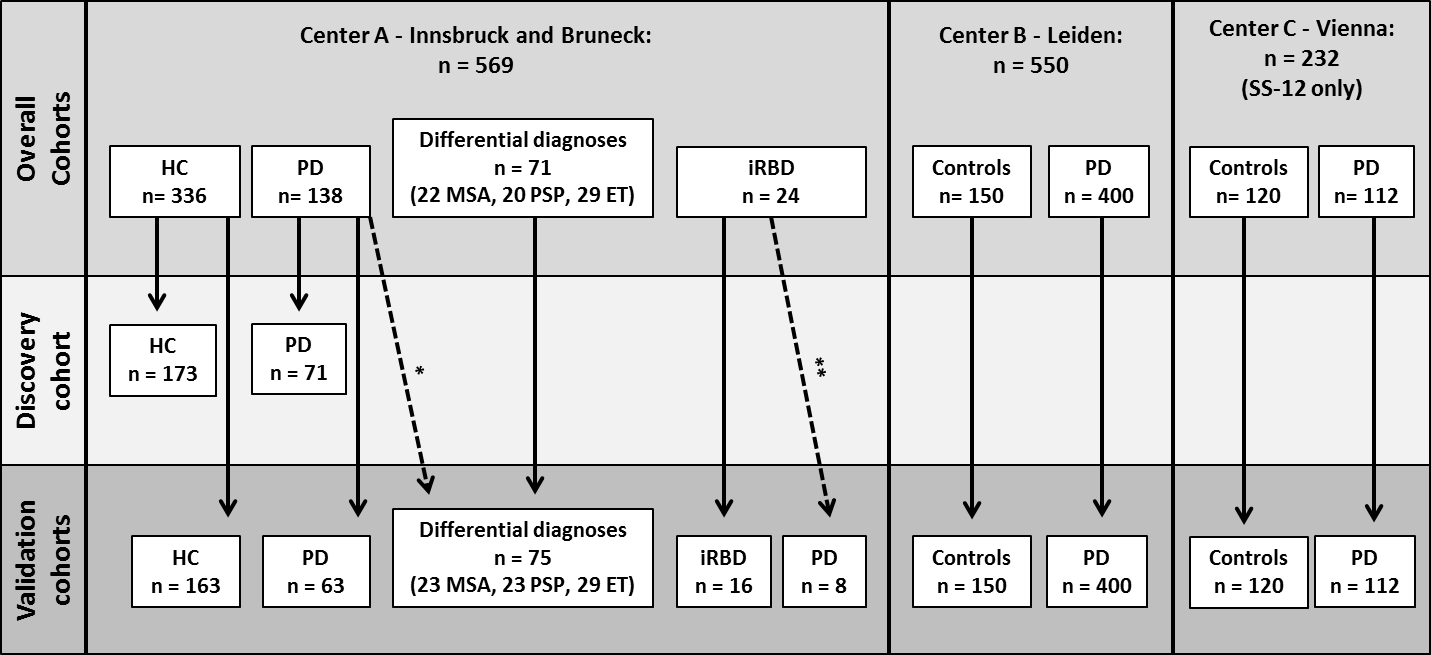
**

**Supplementary Figure 1: Cohorts and study design.**

Abbreviations: ET = Essential Tremor; HC = Healthy Controls; iRBD = idiopathic REM-sleep Behavior Disorder; MSA = Multiple System Atrophy, PD = Parkinson’s Disease; PSP = Progressive Supranuclear Palsy; SS-12 = 12-items Sniffin’ Sticks identification test.

* In center A, PD patients were longitudinally followed-up over ≥24 months to minimize misdiagnosis. Indeed, 4 patients with a diagnosis of PD at the time of olfactory assessment were reclassified as MSA (1 case) or PSP (3 cases).

** Of the 24 iRBD patients longitudinally followed-up over 6 years 8 patients developed PD (±dementia).

**Supplementary Table 1**: Proportion of correctly identified odors in the study groups

|  |  | **Cohort A** | | | | | | | **Cohort B** | | | | **Cohort C** | | | |
| --- | --- | --- | --- | --- | --- | --- | --- | --- | --- | --- | --- | --- | --- | --- | --- | --- |
|  | **Odor** | **HC** | **IPD** | **∆ HC-PD** | **p-Value** | **DD** | **∆ DD-PD** | **p-Value** | **HC** | **IPD** | **∆ HC-PD** | **p-Value** | **Controls** | **PD** | **∆ Controls-PD** | **p-Value** |
| **1** | **Orange** | 90.8 | 75.4 | 15.4 | <0.001 | 84.0 | 8.6 | 0.113 | 54.5 | 85.3 | 30.8 | <0.001 | 90.2 | 76.8 | 13.4 | 0.005 |
| **2** | **Leather** | 80.4 | 44.8 | 35.6 | <0.001 | 76.0 | 31.2 | <0.001 | 55.8 | 88.7 | 32.9 | <0.001 | 84.8 | 61.6 | 23.2 | <0.001 |
| **3** | **Cinnamon** | 78.9 | 31.3 | 47.6 | <0.001 | 72.6 | 41.3 | <0.001 | 28.3 | 71.3 | 43.1 | <0.001 | 72.7 | 43.8 | 28.9 | <0.001 |
| **4** | **Peppermint** | 94.6 | 53.7 | 40.9 | <0.001 | 90.7 | 37.0 | <0.001 | 57.3 | 96.0 | 38.8 | <0.001 | 92.4 | 62.5 | 29.9 | <0.001 |
| **5** | **Banana** | 87.8 | 45.5 | 42.3 | <0.001 | 80.0 | 34.5 | <0.001 | 58.0 | 94.7 | 36.7 | <0.001 | 94.7 | 59.8 | 34.9 | <0.001 |
| **6** | **Lemon** | 60.1 | 38.1 | 22.0 | <0.001 | 49.3 | 11.2 | 0.107 | 34.3 | 58.0 | 23.8 | <0.001 | 60.6 | 33.0 | 27.6 | <0.001 |
| **7** | **Licorice** | 80.4 | 26.9 | 53.5 | <0.001 | 53.3 | 26.4 | <0.001 | 34.0 | 75.3 | 41.3 | <0.001 | 81.1 | 35.7 | 45.4 | <0.001 |
| **8** | **Turpentine** | 71.4 | 32.8 | 38.6 | <0.001 | 68.0 | 35.2 | <0.001 | 36.8 | 38.7 | 1.9 | 0.69 |  |  |  |  |
| **9** | **Garlic** | 82.7 | 59.7 | 23.0 | <0.001 | 82.7 | 23.0 | <0.001 | 64.3 | 83.3 | 19.1 | <0.001 |  |  |  |  |
| **10** | **Coffee** | 81.3 | 34.3 | 46.9 | <0.001 | 69.3 | 35.0 | <0.001 | 45.8 | 84.7 | 38.9 | <0.001 | 96.2 | 68.8 | 27.4 | <0.001 |
| **11** | **Apple** | 49.1 | 14.2 | 34.9 | <0.001 | 41.3 | 27.1 | <0.001 | 15.3 | 48.7 | 33.4 | <0.001 |  |  |  |  |
| **12** | **Clove** | 79.2 | 53.0 | 26.2 | <0.001 | 78.7 | 25.7 | <0.001 | 60.3 | 91.3 | 31.1 | <0.001 | 90.2 | 60.7 | 29.5 | <0.001 |
| **13** | **Pineapple** | 67.3 | 27.6 | 39.7 | <0.001 | 69.3 | 41.7 | <0.001 | 37.5 | 70.7 | 33.2 | <0.001 | 76.5 | 33.9 | 42.6 | <0.001 |
| **14** | **Rose** | 88.4 | 49.3 | 39.1 | <0.001 | 88.0 | 38.7 | <0.001 | 48.3 | 81.3 | 33.1 | <0.001 | 92.4 | 55.4 | 37.0 | <0.001 |
| **15** | **Anise** | 85.1 | 31.3 | 53.8 | <0.001 | 80.0 | 48.7 | <0.001 | 38.0 | 88.7 | 50.7 | <0.001 |  |  |  |  |
| **16** | **Fish** | 94.0 | 60.4 | 33.6 | <0.001 | 94.7 | 34.3 | <0.001 | 69.0 | 99.3 | 30.3 | <0.001 | 89.4 | 53.6 | 35.8 | <0.001 |
|  | **Mean SUM SS-12 (%)** | 81.9 | 45.0 | 36.9 | <0.001 | 75.6 | 30.6 | <0.001 |  |  |  |  | 85.8 | 53.8 | 32.0 | <0.001 |
|  | **Mean SUM SS-16 (%)** | 79.5 | 42.4 | 37.1 | <0.001 | 73.4 | 31.0 | <0.001 | 78.5 | 46.1 | 32.4 | <0.001 |  |  |  |  |

Numbers represent percentage of patients/participants in the various groups correctly identifying one specific odor, the difference between HC and PD patients as well as the difference between patients with one of the DD and PD patients with respective two-sided p-Values calculated with the chi-square test.

**Supplementary table 2: Positive and negative predictive values of the SS-16 and SS-8 in the distinction of PD from DDs according to PD prevalence in two different settings**

|  |  | **Setting 1**  **(PD prevalence 91.8%)** | | **Setting 2**  **(PD prevalence 69.0%)** | |
| --- | --- | --- | --- | --- | --- |
| **Set of odors** | **Cut Off** | **PPV** | **NPV** | **PPV** | **NPV** |
| SS-16 | ≤10 | 97.7% | 46.2% | 89.5% | 81.2% |
|  | ≤9 | 98.3% | 32.1% | 92.1% | 70.4% |
| SS-8 | ≤5 | 97.4% | 50.5% | 88.2% | 83.7% |
|  | ≤4 | 98.7% | 33.1% | 94.0% | 71.0% |

Abbreviations: DD = Differential Diagnoses; PD = Parkinson’s disease; NPV = Negative Predictive Value; PPV = Positive Predictive Value; SS-16 = 16-items Sniffin’ sticks identification test; SS-8 = subscore of the 8 best-discriminating odors (licorice, anise, mint, cinnamon, banana, pineapple, rose, and coffee)

PPVs and NPVs were calculated based on sensitivity and specificity obtained in the validation cohort PD versus DDs differential diagnoses (multiple system atrophy, progressive supranuclear palsy, and essential tremor taken together) and on assumed prevalences of PD among all cases of parkinsonism in different settings – (1) as reported in population-based studies; (2) as reported in a specialized movement disorder service.^16^

**Supplementary table 3: Diagnostic accuracy of the SS-16 and derived sub-scores in the prediction of PD among patient with ≤3 years disease duration in cohort A**

|  | **Set of odors** | **AUC(95%CI)** | **Cut Off** | **Sensitivity**  **(95%CI)** | **Specificity (95%CI)** | **Accuracy**  **(95%CI)** |
| --- | --- | --- | --- | --- | --- | --- |
| **Discovery:**  **PD (n=23) versus HC (n=173)** | SS-16 | 0.92  (0.86-0.97) | ≤10 | 87.0%  (67.0-96.3) | 86.1%  (80.1-90.6) | 86.2%  (80.7-90.4) |
|  | SS-8 | 0.91  (0.83-0.98) | ≤5 | 87.0%  (67.0-96.3) | 84.4%  (78.2-89.1) | 84.4%  (78.2-89.1) |
| **Validation:**  **PD (n=17) versus HC (n=163)** | SS-16 | 0.93  (0.89-0.97) | ≤10 | 88.2%  (64.4-98.0) | 86.5%  (80.3-91.0) | 86.7%  (80.9-90.9) |
|  | SS-8 | 0.95  (0.91-0.98) | ≤5 | 100.0%  (78.4-100) | 84.0%  (77.6-88.9) | 85.6%  (79.6-90.0) |
| **Validation:**  **PD (n=17) versus DD (n=57)** | SS-16 | 0.89  (0.81-0.96) | ≤10 | 88.2%  (64.4-98.0) | 75.4%  (62.8-84.9) | 78.4%  (67.6-86.3) |
|  |  |  | ≤9 | 76.5%  (52.2-91.0) | 84.2%  (72.4-91.7) | 82.4%  (72.1-89.6) |
|  | SS-8 | 0.92  (0.85-0.98) | ≤5 | 100.0%  (78.4-100) | 73.7%  (60.9-83.5) | 79.7%  (69.1-87.4) |
|  |  |  | ≤4 | 82.4%  (58.2-94.6) | 89.5%  (78.5-95.4) | 87.8%  (78.3-93.7) |

Abbreviations: AUC = Area Under the Curve; HC = Healthy Controls; PD = Parkinson’s Disease; 95%CI = 95% Confidence Interval

**References**

1. Reiter E, Mueller C, Pinter B, et al. Dorsolateral nigral hyperintensity on 3.0T susceptibility-weighted imaging in neurodegenerative Parkinsonism. Mov Disord. 2015 Jul;30(8):1068–1076.

2. Scherfler C, Esterhammer R, Nocker M, et al. Correlation of dopaminergic terminal dysfunction and microstructural abnormalities of the basal ganglia and the olfactory tract in Parkinson’s disease. Brain. 2013;136:3028–3037.

3. Mahlknecht P, Seppi K, Stockner H, et al. Substantia nigra hyperechogenicity as a marker for parkinson’s disease: A population-based study. Neurodegener Dis. 2013;12:212–218.

4. Mahlknecht P, Kiechl S, Stockner H, et al. Predictors for mild parkinsonian signs: a prospective population-based study. Parkinsonism Relat Disord. Elsevier Ltd; 2015;21(3):321–324.

5. Gilman S, Wenning GK, Low PA, et al. Second consensus statement on the diagnosis of multiple system atrophy. Neurology. 2008;71(9):670–676.

6. Litvan I, Bhatia KP, Burn DJ, et al. SIC task force appraisal of clinical diagnostic criteria for parkinsonian disorders. Mov Disord. 2003;18(5):467–486.

7. Gibb WR, Lees a J. The relevance of the Lewy body to the pathogenesis of idiopathic Parkinson’s disease. J Neurol Neurosurg Psychiatry. 1988;51(6):745–752.

8. Deuschl G, Bain P, Brin M. Consensus statement of the Movement Disorder Society on Tremor. Ad Hoc Scientific Committee. Mov. Disord. 1998. p. 2–23.

9. Boesveldt S, Verbaan D, Knol DL, et al. A comparative study of odor identification and odor discrimination deficits in Parkinson’s disease. Mov Disord. 2008 Oct 30;23(14):1984–1990.

10. Mahlknecht P, Iranzo A, Högl B, et al. Olfactory dysfunction predicts early transition to a Lewy body disease in idiopathic RBD. Neurology. 2015;84(7):654–658.

11. Hummel T, Konnerth CG, Rosenheim K, Kobal G. Screening of olfactory function with a four-minute odor identification test: reliability, normative data, and investigations in patients with olfactory loss. Ann Otol Rhinol Laryngol. 2001 Oct;110(10):976–981.

12. Tibshiranit R. Regression Shrinkage and Selection via the Lasso. J R Stat Soc. 1996;58(1):267–288.

13. YOUDEN WJ. Index for rating diagnostic tests. Cancer. 1950 Jan;3(1):32–35.

14. Doty RL. Olfaction in Parkinson’s disease and related disorders. Neurobiol Dis. Elsevier Inc.; 2012 Jun;46(3):527–552.

15. Wenning GK, Shephard B, Hawkes C, Petruckevitch a, Lees a, Quinn N. Olfactory function in atypical parkinsonian syndromes. Acta Neurol Scand. 1995;91(4):247–250.

16. Hughes AJ, Daniel SE, Ben-Shlomo Y, Lees AJ. The accuracy of diagnosis of parkinsonian syndromes in a specialist movement disorder service. Brain. 2002 Apr;125(Pt 4):861–870.
